# Misinformation of COVID-19 vaccines and vaccine hesitancy

**DOI:** 10.1038/s41598-022-17430-6

**Published:** 2022-08-11

**Authors:** Sun Kyong Lee, Juhyung Sun, Seulki Jang, Shane Connelly

**Affiliations:** 1 School of Media and Communication, Korea University, 145 Anam-Ro, Seongbuk-Gu, Seoul, 02841 South Korea; 2 Department of Communication, University of Oklahoma, Norman, USA; 3 Department of Psychology, University of Oklahoma, Norman, USA

**Keywords:** Psychology, Human behaviour

## Abstract

The current study examined various types of misinformation related to the COVID-19 vaccines and their relationships to vaccine hesitancy and refusal. Study 1 asked a sample of full-time working professionals in the US (*n* = 505) about possible misinformation they were exposed to related to the COVID-19 vaccines. Study 2 utilized an online survey to examine U.S. college students’ (*n* = 441) knowledge about COVID-19 vaccines, and its associations with vaccine hesitancy and behavioral intention to get a COVID-19 vaccine. Analysis of open-ended responses in Study 1 revealed that 57.6% reported being exposed to conspiratorial misinformation such as COVID-19 vaccines are harmful and dangerous. The results of a structural equation modeling analysis for Study 2 supported our hypotheses predicting a negative association between the knowledge level and vaccine hesitancy and between vaccine hesitancy and behavioral intention. Vaccine hesitancy mediated the relationship between the vaccine knowledge and behavioral intention. Findings across these studies suggest exposure to misinformation and believing it as true could increase vaccine hesitancy and reduce behavioral intention to get vaccinated.

## Introduction

Health misinformation can kill people, both directly and indirectly. During a public health crisis such as the COVID-19 pandemic, exposure to misinformation about the virus’ spread, symptoms of infection, testing opportunities, and prevention methods can lead to erroneous appraisals of the threat, maladaptive coping behaviors, and a range of fatal consequences. More critically, misinformation about the new COVID-19 vaccines and their development process has the potential to induce high levels of vaccine hesitancy in the public^1,2^, preventing vaccination rates sufficient for achieving herd immunity. Due to the high level of uncertainty caused by the pandemic and the relatively fast speed of vaccine development compared to other types of traditional vaccines, the public naturally sought out information to address their vaccine concerns and guide critical decision-making such as whether to get vaccinated or not. However, separating relevant and valid information from false and distorted misinformation about COVID-19 vaccines is difficult when a vast amount of material is being conveyed through media outlets and websites of varying reliability and accuracy. One critically important challenge to obtaining reliable, accurate COVID-19 vaccine information includes the pervasive, unsolicited, and dubious pseudo-news items communicated via online and social media platforms by various types of actors^1^.

Because many people acquire and share news via social media^3^, misinformation can spread quickly through their social networks, and the likelihood of exposure to disinformation and misrepresentations about the vaccine from unverified sources is high. The resulting increase in public anxiety and negative emotional and behavioral responses complicates the process of advising the public through health experts and agencies such as the CDC and WHO. The current research focuses on people’s perceptions of the nature and types of misinformation about COVID-19 vaccines^4^. Additionally, this research examines the relationship between knowledge about COVID-19 vaccines, including its relevant misinformation, and vaccine hesitancy and refusal.

By acknowledging the serious negative impact of health misinformation and its spread, research in infodemiology (i.e., epidemiology of (mis)information^5,6^) identifies the knowledge translation gap between evidence produced by experts and the public’s actual practices and beliefs. Research has identified various quality markers (e.g., source, technical, and content criteria) and their relations with outcome variables (e.g., health-related knowledge and behavioral changes) necessary for effective health communication on the internet. Studies have shown both algorithmic-based correction on Facebook and social correction via anonymous commenters can be effective in reducing beliefs in health misinformation. However, people who believe in conspiracy theories tend to discredit algorithm-based corrections^7,8^.

More research is needed to (a) observe and define the trends and prevalence of health misinformation on various types of social media; (b) understand how relevant misinformation is shared; (c) evaluate the reach and influence of misinformation; and (d) develop and test effective interventions^9,10^. With the aim of increasing scientific knowledge in these four areas—particularly as they pertain to the current global pandemic, this study consisting of two parts examine the nature and content of misinformation about COVID-19 vaccines and their relationships with vaccine hesitancy. Findings can shed light on designing an effective social action campaign for decreasing the public’s vaccine hesitancy, increasing their health information literacy, and buttressing their ability to identify, resist, and refute false claims encountered in-person or online.

## Misinformation on vaccines

Scholars have identified some common misinformation about coronavirus and the pandemic, such as the novel coronavirus being created by the Chinese government as a bioweapon^11^, and that the virus was intentionally created by powerful people^12^. While these types of misinformation emerged during the early period of the COVID-19 pandemic and they are still pervasive, additional rumors and conspiracy theories have emerged about COVID-19 vaccines in particular as governments and scientists invested heavily in capitalizing on existing messenger RNA (mRNA) technology to quickly develop new COVID-19 vaccines. Misinformation, also called as fake news, refers to any inaccurate claims or depictions and disinformation is a subset of misinformation “intended to mislead”^13^ (para. 3). Traditional anti-vaxxer organizations have used mis- and disinformation (including conspiracy theories) as their strategies to persuade people to not get vaccinated; one common piece of misinformation is that vaccines cause Autism, which has been debunked over and over^4,14^.

However, studies show that even after misinformation is corrected, false beliefs can still remain and are difficult to change^11,15,16^. Given that different people use varying criteria to determine the truthfulness of information (e.g., compatibility with existing information, source credibility, others’ beliefs, internal consistency, and supporting evidence), some people are more susceptible to misinformation than others^16^. Also, when misinformation is easy to read or hear, aligns with individuals’ political beliefs^17^, and is not carefully deliberated^18^, people are more likely to believe it as truth.

The anti-vaccine movement uses conspiratorial claims to spread mis/disinformation about COVID-19 and its vaccines^4^. For example, some anti-vaxxers say Bill Gates or other powerful people created the virus as a means to mandate vaccines which would be used to inject people with a microchip so that a global surveillance network can be established. Other anti-vax lobbyists made a false claim about Dr. Anthony Fauci, the head of the National Institute of Allergy and Infectious Diseases of the US National Institutes of Health, indicating that he funded a Wuhan lab so they will “transform an innocuous coronavirus into the lethal and transmissible SARS CoV2 virus.” While these claims are definitely false, they promulgate conspiratorial beliefs, increasing uncertainty and concerns about COVID-19 vaccines and their effectiveness. This potentially affects hesitancy and willingness to get vaccinated in people who may already be hesitant and in those who normally do not question vaccines. Therefore, the first study examined the nature and prevalence of COVID-19 vaccine misinformation circulated among the U.S. public:

**RQ1.** What kinds of misinformation about COVID-19 vaccines do people in the US report being exposed to?

## Misinformation and vaccine hesitancy

Individuals’ decision-making about vaccination is a continuum from active demand for vaccines to complete refusal of all kinds of vaccines^2^. While there are a few external factors influencing vaccine hesitancy such as public health and vaccine policies, health professionals’ recommendations, or communication and media portrayals, Dubé et al.’s model lists six internal factors involved in individuals’ decision-making process: (a) knowledge/information, (b) past experiences, (c) perceived importance of vaccination, (d) risk perception and trust, (e) subjective norms, and (f) religious and moral conviction. As such, vaccine hesitancy is a multifaceted phenomenon influenced by various social, cultural, and political contexts; vaccine hesitant people are a heterogenous group in that they may refuse some vaccines, but agree to others; for this reason, vaccine uptake is not directly related to vaccine hesitancy and it can vary by specific vaccines involved. Newer vaccines, such as those developed for COVID-19, generally bring higher levels of hesitancy^19,20^.

Hornsey et al.^21^ examined the psychological factors influencing rejection of scientific consensus around vaccination. Based on a large sample collected from 24 countries, they found anti-vaccination attitudes were highest among those who (a) were high in conspiratorial thinking, (b) were high in reactance, (c) reported high levels of disgust toward blood and needles, and (d) had strong individualistic/hierarchical worldviews. However, demographic variables (including education) were insignificant or explained minimal amounts of variance. According to this finding, we expect COVID-19 vaccine hesitancy will be higher for those who are misinformed about the virus and its vaccines by the conspiracies related to the pandemic. Certain personality traits such as reactance (or agreeableness) seem also related to vaccine hesitancy (Authors, under review).

Several well-known conspiracy theories are circulated on anti-vaccination websites such as the idea that large pharmaceutical companies and other interested groups exaggerate the benefits of vaccination and hide risks or dangers of vaccines^22^. Regarding COVID-19 vaccines in particular, some new forms of conspiracy theories have emerged. Individual conspiracy beliefs may come from a unitary conspiratorial worldview that believes “it is common for shadowy networks of people with malevolent intentions to execute mass hoaxes on the public in near-perfect secrecy” (p. 308)^21^. Those who feel this way might be motivated to believe conspiracies about science, with negative impacts on vaccination intentions. Previous studies also identified that people’s willingness to endorse conspiracies (e.g., about the assassination of John F. Kennedy or the death of Princess Diana) were generally correlated with a range of “anti-science” attitudes, including anti-vaccination attitudes^23,24^.

From a randomized controlled trial, Loomba et al.^25^ found that exposure to online misinformation about COVID-19 vaccines declined vaccination intent significantly in both the U.S. (6.4% points) and U.K (6.2% points) samples. They also identified some socio-demographic groups distinctly influenced by exposure to misinformation. Females in the US were more impacted by misinformation than males in terms of vaccination intent to protect others, and lower-income groups were more robust to misinformation regarding vaccination intent to protect themselves or others than the highest income group. In the UK, unemployed participants were less likely to lower their vaccine intent upon exposure to misinformation than the employed, and ‘other’ religious affiliations were more robust to misinformation than Christians.

Based on the above research findings, those who are misinformed about the COVID-19 vaccines development and effectiveness, whether via their own conspiratorial beliefs and worldviews^21^, or from exposure to various mis/disinformation about vaccines^23–25^, will be more likely to be hesitant about getting vaccinated against the virus. Thus, we propose the following:

**H1.** Knowledge levels about COVID-19 vaccines are negatively associated with COVID-19 vaccine hesitancy.

Consequently, it is expected that people who are more hesitant to get vaccinated will also be less inclined to actually get vaccinated against the coronavirus. Therefore:

**H2**. COVID-19 vaccine hesitancy is negatively associated with the behavioral intention to get a COVID-19 vaccine.

Finally, the second study examines the following research question about the mediating relationship between vaccine knowledge and vaccination intent by the level of hesitancy:

**RQ2**. Does COVID-19 vaccine hesitancy mediate the influence of knowledge on the behavioral intention to get a COVID-19 vaccine?

## Results for study 1

Study 1, examined the types of COVID-19 vaccine misinformation that people in the US reported being exposed to. Results are summarized in Table 1. Participants indicated the specific nature of misinformation they heard about COVID-19 vaccines. No explicit information was gathered on whether participants believed or agreed with any of the misinformation they reported. Note that out of 505 participants, 17 did not answer the vaccine misinformation questionnaire and responses from a total of 488 participants were coded. Out of 488 participants, 62 participants provided irrelevant responses (e.g., “many”, “false”, “learn about the benefits of COVID-19 vaccination based on what experts currently know”). Out of 426 participants, 207 participants (42.42%) indicated that they did not hear any misinformation related to COVID-19 vaccines. Importantly, it is unclear whether these participants actually did not hear any misinformation about COVID-19, or whether they heard misinformation and believed it to be factual.

**Table 1:** Vaccine misinformation and false claims identified in Study 1.

Coding options	Frequencies	(%)
I didn’t hear any misinformation related to COVID vaccines	207	42.4
Others (not relevant)	62	12.7
COVID-19 vaccines have a microchip	52	10.6
COVID-19 vaccines are dangerous/harmful	36	7.4
COVID-19 vaccines cause death	35	7.2
COVID-19 vaccines cause DNA alteration/generic alterations	26	5.3
COVID-19 vaccines cause COVID-19 disease	23	4.7
COVID-19 vaccines are used for government control	20	4.1
COVID-19 vaccines cause sterilization	17	3.5
COVID-19 vaccines cause other diseases (e.g., Lyme disease)	17	3.5
COVID-19 vaccines are ineffective	15	3.1
Facts related to COVID-19 vaccines were perceived as misinformation	15	3.1
COVID-19 vaccines cause autism	13	2.7
COVID-19 vaccines contain fetal cells/fetus	7	1.4
COVID-19 vaccines are the mark of the beast	4	0.8
COVID-19 vaccines turn people into zombies	2	0.4

The total number does not add up to the sample size of 488 and the sum of percentage scores go over 100% since some participants gave more than one answer about misinformation they heard.

Of the 281 participants who reported hearing COVID-19 vaccine misinformation, 70 participants shared more than one piece of vaccine misinformation. Overall, 14 different types of misinformation were reported. According to the results, the most commonly shared vaccine misinformation was that COVID-19 vaccines contain a microchip to track people. The next commonly shared vaccine misinformation and false claims were that COVID-19 vaccines are dangerous and harmful and that COVID-19 vaccines cause death, respectively.

To further understand what demographic variables significantly differentiated participants who reported hearing no COVID-19 vaccine misinformation from those who reported hearing one or more types of misinformation, a series of logistic regression analyses were performed. Results showed that age (*B* = − 0.02, *p* < 0.05), the number of dependents (*B* = 0.20, *p* < 0.05), and religion specifically Catholic (*B* = 1.18, *p* < 0.01) were significantly associated with hearing no vaccine misinformation, while race, gender, education, marital status, political affiliations, organizational size, sexual orientation, and work industry did not. In other words, people who indicated that they did not hear any misinformation related to COVID-19 vaccines were younger, had more dependents, and Catholic.

## Results for study 2

### Testing of hypotheses 1, 2 and RQ2

**Figure 1:**
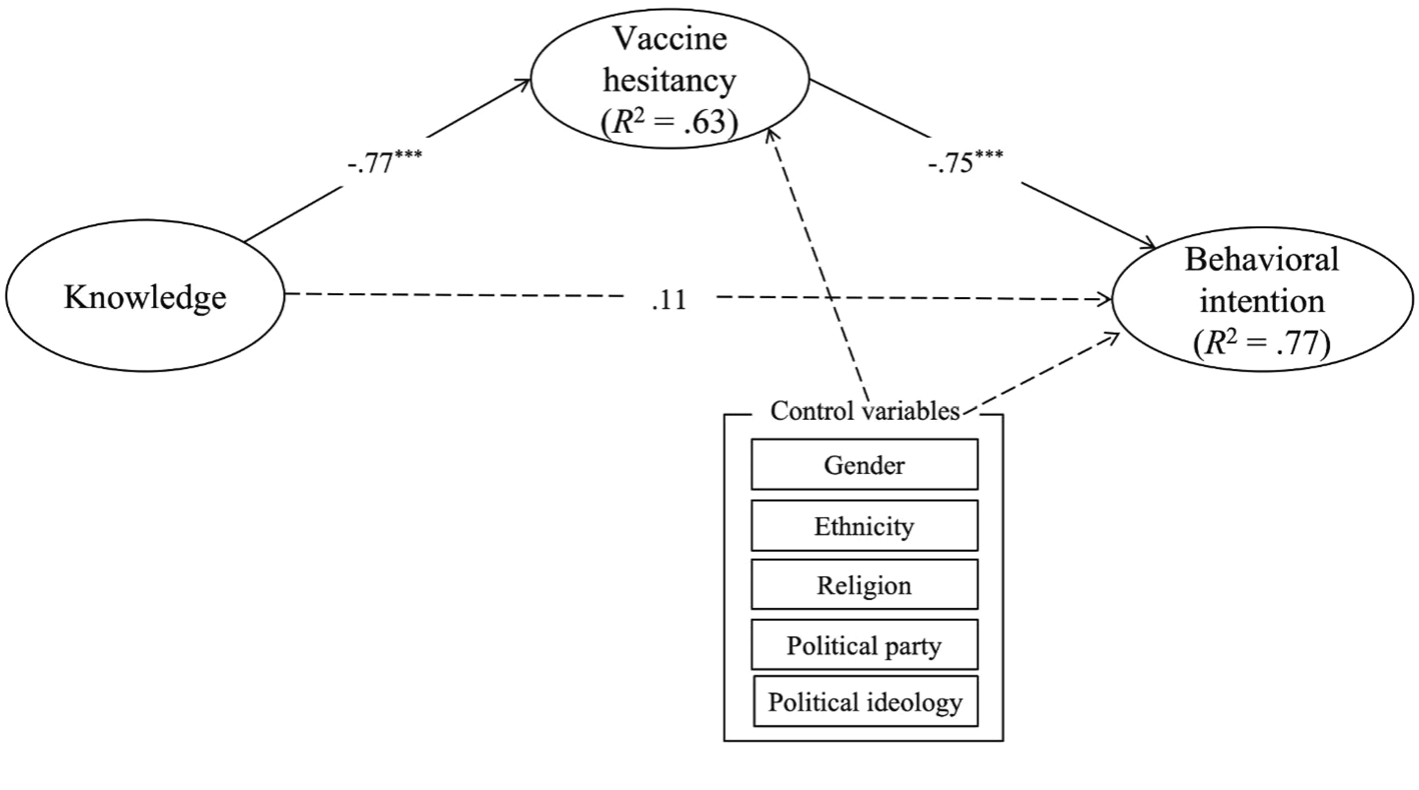
Results of the SEM depicting direct and indirect effects (Study 2). Estimates are indicated in standardized values and the dashed lines indicate non-significant paths. ***p < 0.001.

As indicated in Fig. 1, we examined the associations among knowledge levels about COVID-19 vaccines, COVID-19 vaccine hesitancy, and behavioral vaccination intention for COVID-19. In addition, the mediating role of COVID-19 vaccine hesitancy was investigated. The results of structural equation modeling (SEM) demonstrated that the proposed hypothesized model had a good fit: χ^2^(253) = 655.24, *p* < 0.001, χ^2^/df = 2.59, CFI = 0.92, RMSEA = 0.06, and SRMR = 0.05. The percentage of explained variance in COVID-19 vaccine hesitancy and behavioral intention to get COVID-19 vaccines were 63.0% and 76.5%, respectively.

Figure 1 presents the results for the SEM analysis after controlling for the effects of demographic variables. The results showed that there were no demographic differences in vaccine hesitancy by gender (*p* = 0.15), ethnicity (*p* = 0.65), religion (*p* = 0.37), political party affiliation (*p* = 0.47), and political ideology (*p* = 0.08). On the other hand, demographic differences were seen in behavioral vaccination intention by political ideology (*p* < 0.05) while there were no differences by the other demographic variables: gender (*p* = 0.28), ethnicity (*p* = 0.21), religion (*p* = 0.20), and political party identification (*p* = 0.06). Participants who identified as conservatives were less likely to get vaccinated than those who identified as moderate or liberal.

H1 suggested that knowledge about COVID-19 vaccines would be associated with COVID-19 vaccine hesitancy. We found that individuals’ general knowledge levels about the vaccines had a negative relationship with the levels of vaccine hesitancy for COVID-19 (*b* = − 0.77, *p* < 0.001). The more accurate knowledge participants had about COVID-19 vaccines, the lower-level hesitancy they reported for COVID-19 vaccines, supporting H1.

H2 predicted that COVID-19 vaccine hesitancy would be negatively related to behavioral vaccination intention for COVID-19. The result showed that vaccine hesitancy had a strong negative association with behavioral intention (*b* = − 0.75, *p* < 0.001), which indicated participants who were more hesitant to get COVID-19 vaccines had lower levels of behavioral intention to receive the COVID-19 vaccine in the future. Therefore, H2 was also supported.

We also examined the mediating role of COVID-19 vaccine hesitancy in the relationship between knowledge about COVID-19 vaccines and behavioral vaccination intention (RQ2). The analysis revealed that COVID-19 vaccine hesitancy fully mediated the association. More specifically, as indicated in Fig. 1, the level of knowledge on COVID-19 vaccine was significantly associated with vaccine hesitancy for COVID-19, which in turn significantly affected the behavioral intention to get the vaccines. In addition, knowledge about COVID-19 vaccines was not directly associated with the COVID-19 vaccine intention (*b* = 0.11, *p* = 0.16). The total standardized effect was significant (*b* = 0.68, *p* < 0.001, 95% CI [0.59, 0.77]). Table 2 indicates the results of the mediation analysis of this study.

**Table 2:** Results of mediation analysis.

Variable	Effects on					
	Vaccine hesitancy			Behavioral intentions		
	Direct	Indirect	Total	Direct	Indirect	Total
Knowledge 95% CI	− 0.77*** [− 0.85, − 0.68]	–	− 0.77*** [− 0.85, − 0.68]	.11 [− 0.05, 0.27]	.57*** [0.46, 0.72]	.68*** [0.59, 0.77]

***p < 0.001.

## Discussion

Using qualitative and quantitative data, the current research examined the types of misinformation circulated among the U.S. public about COVID-19 vaccines and how accuracy of people’s knowledge about COVID-19 vaccines related to their vaccine hesitancy and behavioral intentions to get vaccinated. Based on an open-ended question in an online survey of full-time workers, we asked what specific misinformation participants had heard about the COVID-19 vaccines for the qualitative Study 1. Using another online survey of college students, the quantitative Study 2 measured the level of knowledge about COVID-19 vaccines, multiple aspects of vaccine hesitancy, and behavioral intention of vaccination. While many participants in Study 1 (about 41%) reported not having heard of any misinformation, and many participants in Study 2 correctly answered knowledge questions about COVID-19 vaccines (69 to 98% depending on the question), findings of our study still raised some concerns about the prospect of overcoming vaccine misinformation and hesitancy.

Content analysis of the open-ended responses in Study 1 revealed that 57.6% of the participants reported being exposed to one or more pieces of COVID-19 vaccine misinformation. More than half of the 14 unique types of misinformation contained inaccurate information about health effects (e.g., COVID-19 vaccines are dangerous, harmful, or cause DNA alterations) while a third contained conspiratorial misinformation about the vaccines. While a small number of deaths have been linked to COVID-19 vaccines, these were extremely rare cases out of the total vaccinated population (0.002%; CDC, 2021^26^), and the causes of these deaths were not always directly linked to vaccination. While this sample was small, it reflected typical working adults in the US and suggested that a sizable number of people were exposed to misinformation about the purpose and effectiveness of COVID-19 vaccines. In addition, the common type of misinformation about the vaccines seemed consistent in nature with broader conspiracy theories about the pandemic being created by the Chinese government or other powerful people to exert control in the world^11,12^.

The results of Study 1 also resonated with those of Study 2 as significant numbers of participants in Study 2 inaccurately answered knowledge questions about COVID-19 vaccines. For example, over 30% of participants answered “true” for the following false statement: COVID-19 vaccines can give you COVID-19. More than 25% of participants answered “it can be safer to get a disease than to get its vaccine” or “COVID-19 vaccines can cause infertility.” Slightly over 15% of participants answered true for this false statement: COVID-19 mRNA vaccines can alter human DNA. Although not as frequent as the misinformation related to microchip, these aforementioned statements were all identified in Study 1 as types of misinformation participants had heard.

When participants were misinformed about the vaccines and their effects, we expected their inaccurate knowledge would be associated with more vaccine hesitancy and less behavioral intention of getting vaccinated. The results from Study 2 supported those hypotheses and also showed a full mediation of vaccine hesitancy between the level of knowledge and behavioral intention. This finding supported Dubé et al.’s conceptual model^2^ suggesting knowledge as one of the key internal factors of vaccine hesitancy and also confirmed previous findings that conspiratorial thinking was highly associated with anti-vaccination attitudes^4,21–23,25^. The fact that many participants of Study 2 believed misinformation about COVID-19 vaccines as true and correct information as false was alarming and consistent with the high level of vaccine hesitancy and low vaccination rate in the US compared to other countries. As of March 24th, 2022, 65.4% of the U.S. population are fully vaccinated for COVID-19. However, many European countries have over 75% (e.g., Germany, Italy, Portugal) or 70% (e.g., UK, Sweden) vaccination rates, and countries that started vaccination much later than the US and did not have their own vaccine development such as Japan (79%) or South Korea (86%) are catching up quickly^27^. With the US’s close neighbor Canada achieving an 84% vaccination rate, there appears to be some unique vaccine hesitancy factors operating for the U.S. public compared to other developed countries.

Several factors potentially influencing the U.S. exposure to vaccine misinformation and vaccine hesitancy deserve further exploration and study. First, was the anti-vaccination movement more intense in the US media outlets consumed in the US? Were people in the US influenced to a greater extent than those in non-US developed countries by the conspiracy theories spread by anti-vaxxer organizations? Second, were vaccination-related issues more politicized in the US compared to other countries given the timing of the 2020 national election and socio-political and cultural contexts surrounding the election? Other research has shown that vaccine hesitancy is affected by multiple factors including social culture and politics^2,17^. In our Study 2, participants who identified as conservatives were less likely to get vaccinated than those who identified as moderate or liberal. In the midst of the COVID-19 pandemic, the U.S. public experienced a highly contentious presidential election, generating a variety of politically motivated misinformation about COVID-19 and other topics. It seems plausible that the amount of mis/disinformation including those related to the pandemic and vaccines altogether increased immensely due to the competition between then President Donald Trump and Presidential candidate Joe Biden before and during the election period. Use of misinformation as a political weapon might have negatively affected U.S. public health decisions. A recent study showed higher rates of vaccine hesitancy in counties with high support for Donald Trump and this vaccine hesitancy gap widened over the study period (i.e., January 2021 to May 2021)^28^. This research also confirmed the politicized public health recommendations.

### Limitations and future directions

A few limitations are worth noting to caveat our findings and to improve future research on this topic. First, many participants in Study 1 indicated they had not been exposed to COVID-19 vaccine misinformation, which might be a positive sign. However, we had no way to verify whether they truly had not been exposed to misinformation, or whether they were exposed to misinformation and believed it to be factual. Considering the wide range of participants (2.5% to 31.3%) who had incorrect answers on the knowledge test in Study 2, we could not completely ignore the possibility that Study 1 participants (who were slightly older and full-time professionals) were exposed to various types of mis/disinformation without recognizing them as inaccurate.

Second, for both Study 1 and 2, we collected cross-sectional data with which we could not test any causal relationships. Although previous evidence has shown that exposure to and belief in vaccine misinformation significantly affects vaccine hesitancy and uptake^4,21–23,25^, the current study’s data only show correlational relationships between these variables. People involved in the anti-vaccination movement who are highly vaccine hesitant and refusing to get vaccinated might be selectively exposing themselves to more mis- and disinformation due to their beliefs and social interactions within homogeneous networks (i.e., echo-chamber). We think bidirectional relationships would be plausible between knowledge of vaccines and vaccine hesitancy (and refusal) and until longitudinal data are collected, no definite causal relationship can be claimed.

Third, despite the fact that we had a sizable sample in both Study 1 (*n* = 505) and Study 2 (*n* = 441), both samples were somewhat homogeneous in their race/ethnicity and educational composition with over 70% of each sample being white and college-educated. However, the two samples were from different populations as Study 2 sample had close to 70% females and all college students while Study 1 had less than 50% females and consisted mostly of working professionals. Thus, findings from this study cannot be generalized to the U.S. population given the recent Census showing that only 63.7% of the U.S. population are comprised of non-Hispanic Whites^29^. Also, those who were educated in some college or higher level were approximately 62% of the U.S. population; thus, our samples over-represented highly educated people. Research has shown the relationship between education level and vaccine hesitancy in the US was like a U-shape since those with less than high school education and those with PhDs were the most hesitant to get COVID-19 vaccines^28^. King et al. suggested that the actual number of people refusing to get vaccinated would be higher than what their findings showed, given that their sample was more highly educated than the general U.S. population^28^. Similarly, in the current study, the scale of misinformation exposure and its negative relationships with vaccine hesitancy and refusal could be larger than what was reported.

Finally, the current study did not examine other potential reasons for participants believing in mis/disinformation of COVID-19 vaccines other than their inaccurate knowledge. Some of them might have a unitary conspiratorial worldview that made them more vulnerable toward particular mis- and disinformation about vaccines^21^. Others might have had past experiences involving negative health reactions to vaccines, making them hesitant toward other types of vaccines and potentially more susceptible to vaccine mis/disinformation. A future study may adopt an in-depth interview to probe this issue further so we can trace the process of people being exposed to mis/disinformation and believing them as true.

## Method for study 1

### Participants

This data collection took place in March, 2021 upon receiving an approval from the Institutional Review Board (IRB) of the University of Oklahoma. All methods were carried out in accordance with relevant guidelines and regulations of the IRB. At that time, COVID-19 vaccines were being administered to individuals at higher risk such as health care workers, first responders, people who were 65 and older, and people with underlying medical conditions nationwide. Participants were recruited via Mechanical Turk (MTurk) using convenience sampling and a total of 505 individuals participated in this study. Informed consent was obtained from every participant. Of those participants, 46.3% were women, 70.4% were White, 78.5% had a Bachelor’s degree or higher degree, and the average age was 37.54 years (*SD* = 10.30). They all lived in the United States and worked at least 40 h a week.

### Procedure

Participants were asked to answer the following open-ended questionnaire: “Have you heard about any misinformation or false claims related to the COVID-19 vaccines? If so, please write down any false information you have come across.” Once the qualitative responses were collected from participants, they were coded by three researchers who have a Ph.D. in Communication or Psychology. In terms of coding procedures, first, the three coders read the same 40 responses and created their own coding schemes independently. After that, they compared and contrasted their coding schemes with the other two coders’ and generated a common coding scheme along with specific coding rules after an intensive discussion. For example, one coding theme was “COVID-19 vaccines have a microchip.” For that theme, the coders agreed to have a rule that specific responses mentioning a microchip should be coded as category number one. Next, to confirm whether the three coders understood the coding scheme and coding rules in a consistent way, they coded 50 additional responses and again compared their coding. Most were identically coded, though a small number of differences were found. For instance, two coders coded one response as category number seven while the third coded the same response as applicable to category number seven and eight. Those minor differences were resolved after another round of discussion and all coders agreed on the final coding scheme. Based on the agreement, the three coders divided up a total of 488 responses and coded 163 responses independently.

## Method for study 2

### Participants and procedure

To examine the influence of knowledge about the COVID-19 vaccines on vaccine hesitancy, a cross-sectional survey was designed. This online survey was opened for data collection for three months from February 2021 to May 2021 upon receiving an approval from the IRB at the University of Oklahoma. All methods were carried out in accordance with relevant guidelines and regulations of the IRB. During that period of data collection, the COVID-19 vaccines had become available to the public in the United States. Respondents were recruited via convenience sampling from a departmental research pool (i.e., SONA) at the university. Students at the university who were taking courses from the department voluntarily signed up to join the research pool and they could select studies they wanted to participate in. A total of 597 responses were collected for the Study 2 and informed consent was obtained from all participants. Of them, 156 cases were excluded because of incomplete/missing information, leaving 441 responses available for the remaining analyses. Demographics of the participants (*n* = 441) are reported in Table 3.

**Table 3:** Demographic characteristics of respondents in Study 2 (*n* = 441).

Demographics	Number *(n)*	Percentage (%)
**Age**, mean (min–max)	441	20.02 (18–30)
**Gender**		
Female	306	69.4%
Male	133	30.2%
Non-binary or prefer not to answer	2	0.4%
**Ethnicity**		
Caucasian	339	76.9%
Hispanic	33	7.5%
African American	15	3.4%
Native American	16	3.6%
Asian	19	4.3%
Multiracial	11	2.5%
Others	8	1.8%
**Religion**		
Christians	348	78.9%
Jewish	3	0.7%
Muslims	5	1.1%
Buddhists	2	0.5%
Unaffiliated (including atheist/agnostic)	83	18.8%
**Political party affiliation**		
Republican	215	48.8%
Democrat	93	21.1%
Independent	79	17.9%
No preference	47	10.7%
Others	7	1.5%
**Political ideology**		
Conservative	155	35.1%
Moderate	156	35.4%
Liberal	88	20.0%
No preference	40	9.1%
Others	2	0.4%

To be eligible for this study, participants were required to be aged 18 years or older and to have not received a COVID-19 vaccine yet. Participants were also informed about the study’s purpose, procedures, risks and benefits, compensation, voluntary nature of the survey, and confidentiality. Those who consented to participate in this study signed the online informed consent and then were asked to complete the following parts. First, they completed demographic information including age, gender, ethnicity, religion, political party affiliation, and political ideology. Next, they answered questions assessing their knowledge levels about COVID-19 vaccines. Third, they answered questions asking about their hesitancy toward COVID-19 vaccination, and finally, they estimated their behavioral intention to get a COVID-19 vaccine. At the end of the survey, we provided a comprehensive fact sheet about the COVID-19 vaccines. Participants received extra credit for their participation.

### Measures

#### Knowledge about COVID-19 vaccines

To evaluate levels of knowledge (and possible misconceptions) about the COVID-19 vaccines, participants were presented with 10 statements (five true and five false) about the COVID-19 vaccines. An example of a true statement is “With most COVID-19 vaccines, you will need 2 shots to get the most protection” and for a false statement, “COVID-19 vaccines can cause autism”.

Participants were asked whether to the best of their knowledge these statements were true or false. The statements were provided based on the COVID-19 Vaccine Communication Handbook^11^ including widespread myths and anti-vaccination misinformation. The number of correctly answered statements was summed to assess knowledge levels of the COVID-19 vaccines (*M* = 8.35, *SD* = 1.87).

#### COVID-19 vaccine hesitancy (VH)

The Vaccine Hesitancy Scale (VHS)^28^ was used to measure participants’ hesitancy levels to get vaccines in general. To reflect the focus of this study, we specified the items by replacing the word, “vaccines” with “COVID-19 vaccines”, and the word, “childhood” to “me”. For instance, we modified the statement “Childhood vaccines are important for my child’s health” to “COVID-19 vaccines are important for my health”. The VHS includes nine items including seven reversed items in the scoring of the scale (e.g., “COVID-19 vaccines are effective”, and “Getting a COVID-19 vaccine is a good way to protect me from disease”). They were measured on a 5-point Likert-type scale ranging from 1 (strongly disagree) to 5 (strongly agree). As a result of confirmatory factor analysis (CFA), two items (i.e., item 5 and item 9) were excluded due to lower factor loadings (less than 0.4)^30^. The modified scale was valid to conduct further analyses: χ^2^(14) = 44.93, *p* < 0.001, χ^2^/df = 3.21, CFI = 0.99, RMSEA = 0.07, and SRMR = 0.02. The items were averaged with higher scores indicating more hesitancy to get a COVID-19 vaccine (*M* = 2.71, *SD* = 0.93).

#### Behavioral intention (BI) for COVID-19 vaccination

Based on Rothman et al.’s measurement^31^, the behavioral intention to get a COVID-19 vaccine was assessed with two items such as (a) if you were faced with the decision of whether to get the COVID-19 vaccine today, how likely is it that you would choose to get vaccinated? and (b) how likely would you be able to get the COVID-19 vaccine in the future? These items were measured with a 5-point Likert-type scale from 1 (very unlikely) to 5 (very likely). Higher scores on this variable indicate greater intention to receive the COVID-19 vaccine in the future (*M* = 3.30, *SD* = 1.36) (see supplementary information).

### Statistical analysis

Descriptive statistics including mean, standard deviation, skewness, and kurtosis, and intercorrelations of knowledge levels about COVID-19 vaccines, COVID-19 vaccine hesitancy, and behavioral intention to get COVID-19 vaccines are indicated in Table 4. The percentage of true and false responses is indicated in Table 5. The total of COVID-19 vaccine knowledge scores ranged from 2 (6 respondents) to 10 (154 respondents), with a mean of 8.35 (*SD* = 1.87).

**Table 4:** Descriptive statistics of major variables in Study 2.

	1	2	3
1 Knowledge	–		
2 Vaccine hesitancy	− 0.64**	–	
3 Behavioral intention	0.58**	− 0.76**	–
*Mean (SD)*	8.35 (1.87)	2.71 (.93)	3.30 (1.36)
Skewness	− 1.32	0.43	− 0.28
Kurtosis	1.27	− 0.02	− 1.19

**p < 0.01.

**Table 5:** Percentage of true and false responses to general knowledge test about COVID-19 vaccines with correct answers in bold font.

	True (%)	False (%)
Vaccines are the best way to fight preventable infectious diseases [T]	**80.3**	19.7
COVID-19 vaccines can cause autism [F]	6.3	**93.7**
COVID-19 mRNA vaccines can alter human DNA [F]	15.2	**84.8**
Side effects such as fever, chills, tiredness, and headache can occur after getting a COVID-19 vaccine [T]	**97.5**	2.5
Side effects such as fever, chills, tiredness, and headache are transient, and they usually disappear within 24–48 h [T]	**93.0**	7.0
COVID-19 vaccines can cause infertility [F]	25.6	**74.4**
It can be safer to get a disease than to get its vaccine [F]	27.7	**72.3**
With most COVID-19 vaccines, you will need 2 shots to get the most protection [T]	**91.2**	8.8
Keep wearing a mask after you get vaccinated for COVID-19 is safer than not [T]	**79.1**	20.9
COVID-19 vaccines can give you COVID-19 [F]	31.3	**68.7**

Descriptive and correlation analyses were conducted to confirm the normality of the data and to explore the participants’ demographic characteristics and the relationships among all the variables of this study using SPSS 26.0. Before conducting SEM, reliabilities and validities of the measurements were tested. We found that the measurements of this study had robust internal reliabilities as well as convergent and discriminant validities: the Cronbach’s α values were all above 0.80, and the factor loadings of the items of each construct, composite reliabilities (CR) and average variance extracted (AVE) were over 0.60, 0.80, and 0.60, respectively. The outcomes fulfilled the criterion of reliabilities and validities for all the constructs (see Table 6).

**Table 6:** Analysis of internal reliability and validities of the measurements.

Construct	Item	Internal reliability	Convergent and discriminant validity		
		Cronbach’s α	Factor loading	Composite reliability	Average variance extracted
Vaccine hesitancy	VH1	0.94	0.87	0.94	0.70
	VH2		0.88		
	VH3		0.88		
	VH4		0.86		
	VH6		0.82		
	VH7		0.91		
	VH8		0.82		
Behavioral intentions	BI1	0.80	0.68	0.83	0.63
	BI2		0.88		
	BI3		0.94		

Structural equation modeling (SEM) was also performed to examine the measurement model and hypotheses of this study using AMOS 25.0. Maximum likelihood estimation was used to estimate the hypothesized model. To measure the overall fit of the suggested model, the following indices were used^30^: χ^2^/df < 3, the comparative fit index (CFI) > 0.90, the root mean square error of approximation (RMSEA) < 0.07, and the standardized root mean square residual (SRMR) < 0.08. To test the mediating effect of COVID-19 vaccine hesitancy, bootstrapping was applied with 2000 bootstrapped replications and 95% confidence intervals (CIs). All the estimates were indicated in standardized scores. We controlled gender, ethnicity, religion, political party affiliations, and political ideology as they have been significantly related to COVID-19 vaccine hesitancy and behavioral intentions to get the vaccines^32–35^.

## Conclusion

Based on a mixed-method approach, the current study examined various types of misinformation related to COVID-19 vaccines, circulated among the U.S. public, and how accuracy in knowledge of COVID-19 vaccines related to vaccine hesitancy and behavioral intention. The study found from the analysis of open-ended answers that people were exposed to conspiratorial misinformation about COVID-19 vaccines such as the vaccines including a microchip or them being dangerous and harmful, causing death, or altering DNA. The knowledge test utilized in the quantitative study also confirmed many people believed such misinformation related to COVID-19 vaccines as true and inaccurate knowledge seemed to increase their vaccine hesitancy and decrease behavioral intention to get vaccinated. Consistent with previous studies, the findings of this study confirmed the importance of accurate knowledge and influence of misinformation related to vaccine hesitancy and refusal. Public health campaigns and strategies need to be strengthened to combat the conspiracy spread by the anti-vaccine movement and effectively intervene in the circulation of mis/disinformation related to COVID-19 vaccines.

## Supplementary Information

Supplementary Information.
