# Supplementary material for: Misinformation of COVID-19 vaccines and vaccine hesitancy
Source: Sci Rep. 2022 Aug 11;12:13681. doi: 10.1038/s41598-022-17430-6 (PMC9366757; doi:10.1038/s41598-022-17430-6)
Supplement: Supplementary file 1 — Supplementary Information. [file 41598_2022_17430_MOESM1_ESM.docx]

**Appendix**

**Table 1.** Questionnaires for Study 1

| Are you eligible to get a COVID-19 vaccine? | 1. Yes (2) No |
| --- | --- |
| Are you willing to get a COVID-19 vaccine? | 1. Yes (2) No |
| If you are willing to get a COVID-19 vaccine, why? If not, why not? | Open-ended |
| Have you heard about any misinformation or false claims related to COVID-19 vaccines? If so, please write down any false information you have come across. | Open-ended |

**Table 2.** Test items of COVID-19 vaccine knowledge for Study 2

| Items |
| --- |
| 1. Vaccines are the best way to fight preventable infectious diseases. |
| 2. COVID-19 vaccines can cause autism. |
| 3. COVID-19 mRNA vaccines can alter human DNA.  4. Side effects such as fever, chills, tiredness, and headache can occur after getting a COVID- 19 vaccine.  5. Side effects such as fever, chills, tiredness, and headache are transient, and they usually disappear within 24-48 hours. |
| 6. COVID-19 vaccines can cause infertility. |
| 7. It can be safer to get a disease than to get its vaccine. |
| 8. With most COVID-19 vaccines, you will need 2 shots to get the most protection. |
| 9. Keep wearing a mask after you get vaccinated for COVID-19 is safer than not. |
| 10. COVID-19 vaccines can give you COVID-19. |

**Table 3.** (Modified) Vaccine hesitancy scale (VHS) for Study 2

| Items |
| --- |
| *1. COVID-19 vaccines are important for my health. |
| *2. COVID-19 vaccines are effective. |
| *3. Having me vaccinated is important for the health of others in my community. |
| *4. All COVID-19 vaccines offered by the government program in my community are beneficial. |
| 5. New vaccines carry more risks than older vaccines. |
| *6. The information I receive about COVID-19 vaccines from the vaccine program is reliable and trustworthy. |
| *7. Getting COVID-19 vaccines is a good way to protect me from disease. |
| *8. Generally, I do what my doctor or healthcare provider recommends about COVID-19 vaccines for me. |
| 9. I am concerned about serious adverse effects of COVID-19 vaccines. |

*Note*. asterisk (*) indicates reverse-coded item

**Table 4.** (Modified) Behavioral intention to receive COVID-19 vaccines for Study 2

| Items |
| --- |
| 1. How likely would you have the seasonal influenza vaccination sometime soon? |
| 2. If you were faced with the decision of whether to get the COVID-19 vaccine today, how likely is it that you would choose to get the vaccine? |
| 3. How likely would you be able to get the COVID-19 vaccine in the future? |
